# Dr. William Gregory

**Published:** 1858-07

**Authors:** 


AFTER a lingering illness, DR. WILLIAM GREGORY, Professor of Chemistry in the University of Edinburgh, died on the 24th of April. Dr. Gregory stood in the foremost rank of the chemists of his age, having been a private pupil of Liebig, and a translator of several of his works, as well as the author of several standard treatises. His father, James Gregory, was the distinguished Professor of Medicine in the same University, and he himself successively taught chemistry in the Andersonian Institution, Glasgow; King's College, Aberdeen; and in the University of Edinburgh. With the latter institution he had been connected for fifteen years.

